# Sub‐types of insomnia in adolescents: Insights from a quantitative/molecular twin study

**DOI:** 10.1002/jcv2.12167

**Published:** 2023-05-06

**Authors:** Juan J. Madrid‐Valero, Frühling Rijsdijk, Saskia Selzam, Helena M. S. Zavos, Melanie Schneider, Angelica Ronald, Alice M. Gregory

**Affiliations:** ^1^ Department of Health Psychology Faculty of Health Sciences University of Alicante Alicante Spain; ^2^ Social, Genetic and Developmental Psychiatry Centre Institute of Psychiatry, Psychology & Neuroscience King's College London London UK; ^3^ Department of Psychology Institute of Psychiatry, Psychology & Neuroscience King's College London London UK; ^4^ Department of Psychology Goldsmiths, University of London London UK; ^5^ Department of Psychological Sciences Birkbeck, University of London London UK

**Keywords:** heritability, insomnia, polygenic scores, sleep duration, sub‐types, twins

## Abstract

**Background:**

Insomnia with short sleep duration has been postulated as more severe than that accompanied by normal/long sleep length. While the short duration subtype is considered to have greater genetic influence than the other subtype, no studies have addressed this question. This study aimed to compare these subtypes in terms of: (1) the heritability of insomnia symptoms; (2) polygenic scores (PGS) for insomnia symptoms and sleep duration; (3) the associations between insomnia symptoms and a wide variety of traits/disorders.

**Methods:**

The sample comprised 4000 pairs of twins aged 16 from the Twins Early Development Study. Twin models were fitted to estimate the heritability of insomnia in both groups. PGS were calculated for self‐reported insomnia and sleep duration and compared among participants with short and normal/long sleep duration.

**Results:**

Heritability was not significantly different in the short sleep duration group (*A* = 0.13 [95%CI = 0.01, 0.32]) and the normal/long sleep duration group (*A* = 0.35 [95%CI = 0.29, 0.40]). Shared environmental factors accounted for a substantial proportion of the variance in the short sleep duration group (*C* = 0.19 [95%CI = 0.05, 0.32]) but not in the normal/long sleep duration group (*C* = 0.00 [95%CI = 0.00, 0.04]). PGS did not differ significantly between groups although results were in the direction expected by the theory. Our results also showed that insomnia with short (as compared to normal/long) sleep duration had a stronger association with anxiety and depression (*p* < .05)—although not once adjusting for multiple testing.

**Conclusions:**

We found mixed results in relation to the expected differences between the insomnia subtypes in adolescents. Future research needs to further establish cut‐offs for ‘short’ sleep at different developmental stages and employ objective measures of sleep.

**Key points**

- This study sheds light on the development and progression of insomnia and supports the use of programs to prevent the progression and worsening of insomnia at early stages of life.
- Our results also highlight a major role of non‐shared environmental factors in explaining individual differences for insomnia among short sleepers.
- Results from this study are potentially of interest for both basic research and clinical practice since sleep is a modifiable behaviour.
- Our results could also be important for nosology—helping to refine the categorization of insomnia.

## INTRODUCTION

Insomnia is one of the most common sleep disorders (Morin et al., 2015). Prevalence of insomnia varies across studies ranging from 6% to 33% depending on the criterion used (Bixler et al., 2002; Ohayon, 2002; Ohayon & Sagales, 2010). Insomnia has been extensively studied in adults, however, insomnia in adolescents is poorly characterized, underrecognized, under‐diagnosed, and under‐treated despite its high prevalence (de Zambotti et al., 2018). In adults, a theory about two different types of insomnia (i.e., insomnia with short sleep duration vs. insomnia with longer sleep duration) has been proposed. This theory states that these subtypes may differ in terms of their aetiologies, have different characteristics and are associated with different phenotypes. Specifically, it has been proposed that insomnia with short sleep duration (defined by the authors as <6 h) reflects a biological vulnerability (e.g., genetic predisposition), and is characterised by physiological hyperarousal, impaired neurocognitive functioning, increased risk of cardiometabolic morbidity and it is likely to persist. In contrast, insomnia with longer sleep duration is not associated with physiological hyperarousal, but may involve sleep misperception, anxious‐ruminative profile and is more likely to remit. Insomnia is related to a wide variety of negative consequences such as depression, anxiety and cardiometabolic morbidity and mortality among others. This theory states that there are differences between the subtypes when it comes to associated morbidity such as depression (Vgontzas et al., 2013).

The idea that there are two different types of insomnia based on sleep duration and that one subtype has a greater ‘biological’ component as compared to the other has not been widely tested. As the authors state in their manuscript (Vgontzas et al., 2013), future research should examine the underlying genetics of this theory. Both twin studies and genome‐wide association studies (GWAS) have shown that insomnia is substantially influenced by genetic factors (Gregory et al., 2016; Hammerschlag et al., 2017; Hublin et al., 2011; Jansen et al., 2019; Madrid‐Valero et al., 2021; Taylor et al., 2015). In GWAS studies, a substantial genetic correlation between sleep duration and insomnia (−0.47) was found (Hammerschlag et al., 2017; Jansen et al., 2019) with overlapping loci (Jansen et al., 2019). This suggests an overlap between risk genes for insomnia and short sleep duration. Furthermore, a recent meta‐analysis showed that genetic and environmental influences on sleep duration change across the life span (Kocevska et al., 2021). However, there are no studies that have tested the theory by considering insomnia in those with short and normal/long sleep duration, from a quantitative/molecular genetics perspective. Using such approaches, we can examine whether the heritability of insomnia symptoms is higher in participants with short sleep duration as compared to those with normal/long sleep duration. Furthermore, the use of polygenic scores (PGS) allows us to test genetic risk for insomnia (and also short sleep duration which is genetically associated with insomnia) in both groups.

The focus of this study is on participants at a young age (adolescents) since this could shed light on the development and progression of insomnia. Programs could be applied to prevent the progression and worsening of insomnia at early stages of life which could have an impact on the conception and treatment of insomnia in adolescents. Some authors have described factors contributing to sleep difficulties in adolescence as the ‘perfect storm’. During this developmental stage the maturation of the regulatory sleep system in combination with psychological and social pressures could affect sleep (e.g., resulting in shorter sleep duration and sleep disturbances including insomnia) (Crowley et al., 2018). A previous study has shown that insomnia is associated with cortical hyperarousal as early as adolescence (Fernandez‐Mendoza, Li, et al., 2016). These factors are likely influenced by both genetic and environmental factors and knowing more about these influences could help increase understanding of the aetiology of insomnia during adolescence. Although the literature about insomnia with short sleep duration is scarce in adolescents, some studies have revealed that insomnia with short sleep duration is associated with systemic inflammation and an increased risk of depression (Fernandez‐Mendoza, Calhoun, et al., 2016; Fernandez‐Mendoza et al., 2017). This study aims to increase understanding of insomnia with short sleep versus. insomnia with normal/long sleep duration from different perspectives: (1) from a quantitative genetic perspective, testing the heritability of insomnia symptoms in two groups of participants (i.e., those with short vs. those with normal/long sleep duration); (2) from a molecular genetic perspective, examining PGS for insomnia symptoms and sleep duration in subjects with short and with normal/long sleep duration; (3) comparing those with insomnia and short versus normal/long sleep on a wide variety of traits previously associated with insomnia symptoms. We also performed exploratory analyses for other phenotypes such as psychotic‐like symptoms and educational achievement in order to generate additional information relevant to this theory.

## METHODS

### Participants

The sample comprised 4000 twin pairs from the Twins Early Development Study (TEDS), which is a community‐based, longitudinal study of twins born in England and Wales between 1994 and 1996 (Haworth et al., 2013; Rimfeld et al., 2019). This sample is reasonably representative of the general population (Kovas et al., 2007). Zygosity was established using either DNA testing or a questionnaire which has shown an accuracy of over 95% (Price et al., 2000). TEDS and consent procedure were approved by the King's College London ethics committee (ref: PNM/09/10‐104).

This work focuses on data collected at age 16 (the wave at which insomnia symptoms and sleep length were assessed). In this wave 19, 874 families were contacted and invited to participate and 47.3% of these families provided data. Differences between participating and non‐participating families have been reported elsewhere (Zavos et al., 2016). Analyses here focused on a study within this wave of data collection called LEAP‐1 (including 4000 families who provided data about sleep). The sample was 46.0% male and 35.4% monozygotic.

### Measures

#### Insomnia

Insomnia was measured using the Insomnia Severity Index (ISI). The ISI has seven items enquiring about symptoms of insomnia over the previous month. Scores range from 0 to 28 (Bastien et al., 2001)—with a higher score reflecting greater insomnia symptoms. This questionnaire has shown adequate psychometric properties and there is a cut‐off of eight points for the ISI questionnaire to categorise participants at risk of insomnia (Morin et al., 2011). Cronbach's alpha in our sample was .89.

#### Sleep duration

Sleep duration was assessed using the question ‘During the past month, how many hours of actual sleep did you get at night? (This may be different than the number of hours you spend in bed)’. This item was taken from the Pittsburgh Sleep Quality Index questionnaire (Buysse et al., 1989). The sleep duration categorization was modified from the original theory proposed by Vgontzas et al. (2013) who focused on <6 h (of note, the recommended sleep length for most adults is between 7 and 9 h (Hirshkowitz et al., 2015)). The modification occurred because the work reported here does not focus on adults, but on 16‐year‐old participants. Guidelines recommend that most teenagers of this age require 8–10 h of sleep each night (Hirshkowitz et al., 2015)—meaning that less than 7 h is considered to be short sleep for most participants of this age group. Data were therefore categorised to focus on those who slept for less than 7 h (short sleepers) versus those who slept for 7 h or more (normal/long sleepers) which is consistent with previous studies performed in adolescence (Fernandez‐Mendoza, Calhoun, et al., 2016). Sensitivity analyses were not run using the more restrictive criterion due to the small number of participants who slept less than 6 h (*n* = 511 participants).

#### Anxiety

Anxiety was measured using the emotional symptoms/anxiety domain of the Strengths and Difficulties Questionnaire (SDQ). This is a five‐dimension questionnaire assessing the core domains of psychopathology in children and adolescents (Goodman, 1997). For this study, we focused on the anxiety subscale (five items; theoretical range 0–10). The SDQ has shown good psychometric properties (Stone et al., 2010). The Cronbach's alpha for the anxiety sub‐scale in this sample was .69.

#### Anxiety sensitivity

Anxiety sensitivity was measured using the Children's Anxiety Sensitivity Index (CASI) (Silverman et al., 1991). This questionnaire has 18 items (theoretical range 0–36) referring to fear of the physical symptoms of anxiety. The CASI has good validity and internal consistency (Muris et al., 2001; Silverman et al., 1991). In this sample the Cronbach's alpha was .86.

#### Depression

Depression was measured using the Short Mood and Feelings Questionnaire (SMFQ). The SMFQ questionnaire consist of 13 items with responses for each from 0 (not true) to 2 (true) and these scores can yield a total score ranging from 0 to 26. These questions assess key symptoms of depression and focus on the previous 2 weeks (Angold et al., 1995). SMFQ has shown adequate psychometric properties (Kent et al., 1997; Sharp et al., 2006). Cronbach's alpha in the current sample was .88.

#### Psychotic‐like symptoms

Psychotic‐like symptoms were measured using the Psychotic Like Symptom Questionnaire (Zammit et al., 2011). This measure consists of 29 questions (theoretical range 0–91) inquiring about hallucinations (visual and auditory); delusions (delusions of being spied on, persecution, thoughts being read, reference, control, grandiose ability); and experiences of thought interference (thought broadcasting, insertion and withdrawal). At least 5 responses were required to calculate the total score. Cronbach's alpha in this sample was .75.

#### Educational achievement

General Certificate of Secondary Education (GCSE) exam results were obtained from twins themselves or from their parents via questionnaires sent over mail or via telephone. GCSEs are UK‐wide standardized examinations taken at age of 16 at the end of compulsory education. Here we focus on the mean grade for English, Maths and Science (taken by nearly all twins in England and Wales) and scores could range from 4 to 11.

#### Polygenic scores

PGS for individuals are the sum of the number of trait‐associated alleles genetic variant weighted by their association effect size based on summary statistics from the largest GWA study available for insomnia (Jansen et al., 2019) and the GWA study by Jones et al. (2016) for sleep duration (please note that a higher score on this measure is associated with longer sleep). PGS were calculated using LDpred which re‐weights the variants effect sizes using a prior on their effect size (based on the heritability and assumed fraction of causal markers that influence the trait), adjusting for the linkage disequilibrium in the sample (Vilhjálmsson et al., 2015). Details about the genotyping data can be found elsewhere (https://www.teds.ac.uk/datadictionary/studies/dna.htm). In TEDS there have been five phases of genotyping data since 1998 which has contributed to the ‘genotypic sample’ where genome‐wide PGS were calculated. DNA was collected from cheek swabs during phases 1–4, and from saliva samples during phase 5. The Affymetrix platform was used for the cheek swab samples from phases 1–4 (AffymetrixGeneChip 6.0 SNP arrays). The Illumina Human OEE platform was used for the saliva samples from phase 5 (using OmniExpressExome‐8v1.2 arrays). The OEE platform was also used for some cheek swab samples from earlier phases (see https://www.teds.ac.uk/datadictionary/studies/dna.htm#oee). Information regarding exclusions can be found on the TEDS data dictionary website (https://www.teds.ac.uk/datadictionary/studies/dna.htm). Broad exclusions were made on the basis that parent‐reported ethnic origin was not white or that serious medical conditions and/or perinatal complications had been reported. The TEDS genotypic sample was composed of data from both the Affymetrix and OEE platforms, which were combined, and quality control procedures were applied (these methods are described in detail in S1 Methods, Supplementary Methods, Selzam et al., 2018). The initial sample comprised 11,869 samples. From this initial sample, 1523 samples were removed due to possible non‐European ancestry, heterozygosity anomalies, genotype call rate <0.98. Regarding the PGS in our study just one member of the twin pair provided data for PGS. All PGS were statistically adjusted for the first ten principal components, chip and plate using the regression method, and were z‐standardized (mean = 0, SD = 1) to avoid potential effects due to population stratification and genotyping. Three thresholds for PGS were used (*p* = .01, .3 and 1), which represent an assumption on the fraction of genetic markers that are causally influencing the trait. For example, *p* = 1 represents the assumption that all genetic variants contribute to trait development. Analyses were performed using these three thresholds to capture possible differences due to the threshold used to calculate the PGS. Both PGS (insomnia and sleep duration) were significantly associated with our insomnia symptoms variable for two of the three thresholds (threshold = 0.01, *p* = .957, *p* = .794; threshold = 0.3, *p* < .01, *p* = .016; threshold = 1, *p* < .01, *p* = .019; for insomnia and sleep duration, respectively) and *R* ^2^ was lower than 1% in both cases.

### Statistical analyses

This study was preregistered on the Centre for Open Science Website (https://osf.io/udsw3). In order to test the hypotheses, several analyses were carried out.

#### Twin analyses

First, quantitative genetic analyses were run. Making use of the difference between monozygotic (MZ) twins (who share 100% of their DNA) and dizygotic (DZ) twins (who share on average 50% of their segregating genes), the variance of a phenotype can be decomposed into genetic and environmental factors (Knopik et al., 2017). The components explaining the variance are: additive genetic influences (*A*; the sum of allelic effects across all loci); and environmental influences—both shared (*C*; influences that make twin pairs raised in the same family similar to each other) and non‐shared (*E*; effects that make family members less alike—a variance component that also includes measurement error) (Verweij et al., 2012).

The univariate model for insomnia using these data, showing that 41% of the variance for insomnia was accounted for by genetic factors, has been reported elsewhere (Madrid‐Valero, Ronald, et al., 2020). In order to test the hypothesis that the heritability of insomnia differs between those with short sleep duration and those with normal/long sleep duration, we fitted a model which allows us to estimate the heritability of insomnia for two groups differing in sleep length. This model is similar to a sex‐limitation model where the heritability of the phenotype is estimated for men and women separately (Neale et al., 2006)—but here we examined the heritability of insomnia symptoms for participants with short sleep duration and normal/long sleep duration. In a sex‐limitation model, there are no MZ twins discordant for sex whereas in this adapted model, it was possible to have discordant MZ twins (where one twin within a pair reported short sleep duration but the other did not). For this specific model six groups were therefore used: (1) MZ twins concordant for short sleep duration [*N* = 151 twin pairs]; (2) MZ twins concordant for normal/long sleep duration [*N* = 879 twin pairs]; (3) MZ twins discordant for sleep duration [*N* = 337 twin pairs]; (4) DZ twins concordant for short sleep duration [*N* = 240 twin pairs]; (5) DZ twins concordant for normal/long sleep duration [*N* = 1481 twin pairs]; (6) DZ twins discordant for sleep duration [*N* = 766 twin pairs]. Different models focusing on qualitative and quantitative differences were fitted. Qualitative differences refer to differences in the *types* of genetic or environmental influences on a trait. Quantitative differences refer to significant differences between groups in terms of the *magnitude* of the influences: Three models were fitted (1) the full heterogeneity model, where there were qualitative and quantitative differences between the two sleep duration groups; (2) the quantitative heterogeneity model where there were quantitative (but not qualitative) differences between the sleep duration groups; (3) the homogeneity model for sleep duration (where qualitative and quantitative differences between the groups were not allowed). Nested models (i.e., AE, CE, E) were also fitted to check if one (or two) components could be dropped without a significant decrease in model fit.

The fits of the different models and submodels were checked using the likelihood‐ratio chi‐square test and the Akaike's information criterion (Akaike, 1987). Age and sex were used as covariates.

#### PGS analyses

PGS were also compared between insomnia groups and participants with short sleep duration (<7 h/night) and normal/long sleep duration (≥7 h of sleep). Four groups were created to compare PGS for insomnia and sleep duration: (1) short sleep duration with ISI scores >8 (referred to hereonin as ‘insomnia’) [*N* = 110]; (2) short sleep duration with ISI scores under 9 (referred to hereonin as ‘no insomnia’) [*N* = 70]; (3) normal/long sleep duration and insomnia [*N* = 391]; (4) normal/long sleep duration and no insomnia [*N* = 2495]. ANOVA and ANCOVA tests including age and sex tested differences among groups as well as *T*‐tests to compare PGS between group 1 and group 3.

#### Correlations

Correlation models were fitted to examine the association between insomnia symptoms and psychological variables. These models allow us to estimate the magnitude of the correlations between insomnia symptoms and associated variables for the different sleep duration groups while controlling for the relatedness of the sample. In order to check whether these correlations significantly differ between groups, we equated the correlations of the three groups (i.e., 1‐concordant for short sleep duration; 2‐concordant for normal/long sleep duration; 3‐discordant for sleep duration). We looked to see whether the decrease in model fit was significant. The logic of this approach is as follows: if the model with fewer parameters (that in which the correlations among the different groups are equated) does not show a significant decrease in fit then there are no statistically significant differences among these correlations and it can be assumed that correlations between insomnia and the analysed variable have a similar magnitude across groups. In other words, the association between insomnia and the analysed variable is not weaker or stronger depending on sleep duration.

#### Deviation from the protocol

Initially we proposed to run Defries‐Fulker extreme analyses, however these were not performed since most of the sample had an insomnia score lower than 9 (87%) which is considered the least restrictive cut‐off for the ISI. Therefore, the sample size at the extremes was limited to perform this analysis. Similarly, participants with high/low levels of insomnia symptoms were not selected from the upper and lower 15% of the distribution. Instead, they were selected using the previously established cut‐off from the ISI which we considered to be a more valid approach (Morin et al., 2011). While we did not initially propose PGS analyses focusing on sleep duration (described above). These were added following feedback obtained during the review process.

## RESULTS

### Twin modelling of insomnia

The best fit was provided by the quantitative heterogeneity model—where the magnitude of the ACE estimates for insomnia symptoms could differ for those with short and normal/long sleep (Table 1)—but there were no qualitative differences. Twin correlations are displayed in Table 2.

**TABLE 1:** Model fit statistics.

Model	Model for comparison	Parameters	df	−2LL	AIC	Diff −2LL	Diff df	*p*
Full‐heterogeneity (quantitative and qualitative differences)	/	11	7660	24,068.75	24,090.75			
Quantitative‐heterogeneity	Full‐heterogeneity (quantitative and qualitative differences)	**8**	**7663**	**24,071.33**	**24,087.33**	**2.57**	**3**	**.46**
Homogeneity	Quantitative‐heterogeneity	4	7667	24,906.64	24,914.44	835.11	4	<.001

*Note*: The bold text identifies the best fitting model.

Abbreviations: AIC, Akaike’s information criterion; df, degrees of freedom; −2LL, negative 2 log likelihood.

**TABLE 2:** Twin pair correlations (with 95% confidence intervals) for insomnia in each group defined by sleep duration.

	Correlation
MZ concordant short sleep duration (*N* = 151)	0.39 (0.24, 0.51)
MZ concordant normal/long sleep duration (*N* = 879)	0.38 (0.33, 0.43)
DZ concordant short sleep duration (*N* = 240)	0.22 (0.09, 0.33)
DZ concordant normal/long sleep duration (*N* = 1481)	0.12 (0.07, 0.17)
MZ discordant sleep duration (*N* = 337)	0.18 (0.08, 0.28)
DZ discordant sleep duration (*N* = 766)	0.14 (0.07, 0.21)

*Note*: Correlations regardless sleep duration were 0.42 (0.38, 0.45) for MZ twins and 0.21 (0.19, 0.23) for DZ twins. *N* refers to number of twin pairs.

Abbreviations: DZ, dizygotic; MZ, monozygotic.

For the short sleep duration group, there was a low, albeit significant, estimate for additive genetic factors (*A* = 0.13 [95% confidence intervals (CI) = 0.01, 0.32]). For the short sleep duration group common shared environmental factors accounted for a significant proportion of the variance on insomnia (*C* = 0.19 [95% CI = 0.05, 0.32]). The rest of the variance was explained by non‐shared environmental factors (*E* = 0.68 [95% CI = 0.57, 0.78]).

Regarding the normal/long sleep duration group, estimates from the quantitative heterogeneity model showed that additive genetic factors explain a substantial proportion of the variance on insomnia (*A* = 0.35 [95% CI = 0.29, 0.40]). Of note the CI for the genetic influences on insomnia overlapped for the two sleep length groups. For the normal/long sleep length group common shared environmental factors had a negligible impact (*C* = 0 [95% CI = 0, 0.04]). Finally, non‐shared environmental influences had a similar influence (*E* = 0.65 [95% CI = 0.60, 0.70]) as compared to the short‐sleep duration group (Table 3). Unstandardized estimates are displayed in Table S1. The variance for insomnia symptoms was greater for those with short sleep duration as compared to normal/long sleep duration.

**TABLE 3:** Genetic and environmental influences on insomnia split into short and normal/long sleep duration groups.

Model	Short sleep duration			Normal/long sleep duration		
	*A*	*C*	*E*	*A*	*C*	*E*
Full‐heterogeneity (qualitative and quatitative differences)	0.35 (0.05, 0.51)	0.04 (0, 0.27)	0.61 (0.49, 0.75)	0.35 (0.29, 0.40)	0 (0, 0.01)	0.65 (0.60, 0.70)
Quantitative‐heterogeneity	**0.13** (**0.01, 0.32)**	**0.19** (**0.05, 0.32)**	**0.68** (**0.57, 0.78)**	**0.35** (**0.29, 0.40)**	**0** (**0, 0.04)**	**0.65** (**0.60, 0.70)**
Homogeneity	0.42 (0.31, 0.45)	0 (0, 0.08)	0.58 (0.55, 0.63)	0.42 (0.31, 0.45)	0 (0, 0.08)	0.58 (0.55, 0.63)

*Note*: *A*, additive genetic influences; *C*, common‐shared environmental influences; *E*, non‐shared environmental influences. The bold text identifies the best fitting model.

Nested models (i.e., AE and CE) were also tested for the quantitative heterogeneity model but dropping parameters resulted in a significant decrease in fit (*p* < .02), suggesting that neither of these parameters could be dropped from the model.

### Polygenic scores

PGS means for insomnia are displayed in Table 4 for the four groups. PGS gradually increased from group 4 (normal/long sleep duration and no insomnia) to group 1 (short sleep duration and insomnia) except using the 0.01 threshold where group 2 was the group with the highest PGS mean. We found small differences in line with the hypothesis that insomnia with short sleep duration is more influenced by genetic factors (PGS were slightly higher in group 1 than group 3), although the ANOVA showed that there were no statistically significant differences among these four groups for any of the three used thresholds to calculate PGS for insomnia (threshold = 0.01, *p* = .498; threshold = 0.3, *p* = .101; threshold = 1, *p* = .102). These results also remained non‐significant in an ANCOVA controlling for sex and age (*p* > .05). Additionally, *T*‐tests revealed no differences between group 1 (short sleep duration and insomnia) and group 3 (normal/long sleep duration and insomnia); (threshold = 0.01, *p* = .569; threshold = 0.3, *p* = .180; threshold = 1, *p* = .210). We did not correct for multiple testing because none of the tests reached the 0.05 significance level.

**TABLE 4:** Polygenic scores (PGS) by group and PGS *p*‐value threshold for insomnia and sleep duration.

	Insomnia PGS (SD) (Threshold = 1)	Insomnia PGS (SD) (Threshold = 0.3)	Insomnia PGS (SD) (Threshold = 0.01)
Short sleep duration—Insomnia (*N* = 110)	0.131 (1.0)	0.137 (1.0)	−0.006 (1.0)
Short sleep duration—No insomnia (*N* = 70)	0.066 (1.2)	0.058 (1.2)	0.128 (1.0)
Normal/long sleep duration—Insomnia (*N* = 391)	−0.008 (1.0)	−0.011 (1.0)	−0.067 (1.0)
Normal/long sleep duration—No insomnia (*N* = 2495)	−0.072 (1.0)	−0.073 (1.0)	−0.022 (1.0)

	Sleep duration PGS (SD) (Threshold = 1)	Sleep duration PGS (SD) (Threshold = 0.3)	Sleep duration PGS (SD) (Threshold = 0.01)
Short sleep duration—Insomnia (*N* = 110)	−0.123 (1.0)	−0.118 (1.0)	0.041 (1.0)
Short sleep duration—No insomnia (*N* = 70)	−0.226 (1.0)	−0.248 (1.0)	−0.135 (1.1)
Normal/long sleep duration—Insomnia (*N* = 391)	−0.056 (1.1)	−0.057 (1.1)	0.052 (1.1)
Normal/long sleep duration—No insomnia (*N* = 2495)	0.048 (1.0)	0.050 (1.0)	0.019 (1.0)

*Note*: PGS are *z* standardised scores which reflect the genetic risk of insomnia/sleep duration that is captured by the PGS. Threshold refers the assumption on the fraction of causal genetic markers that are causally influencing the trait. Short sleep duration refers to those who self‐report for sleeping <7 h a night. Insomnia refers to those who score 8 or more on the ISI. PGS was calculated from information reported in the largest study to date focusing on the genetics of insomnia (Jansen et al., 2019) and sleep duration (Jones et al., 2016). There were no statistically significant differences among groups. In PGS for insomnia the higher the score is the higher the risk of insomnia whereas in PGS for sleep duration the higher the score is the lower the risk of having short sleep duration. *N* refers to number of participants.

Since insomnia and short sleep duration are highly genetically correlated, we also tested the genetic risk for short sleep duration among the different groups. Small differences in line with this hypothesis were also found using PGS for sleep duration as an additional analysis. PGS for sleep duration were slightly lower in group 1 than group 3 (the higher the PGS score the lower the risk of having short sleep duration; Table 4), the ANOVA showed significant differences among these four groups in two of the three used thresholds (threshold = 0.01, *p* = 0.558; threshold = 0.3, *p* = .012; threshold = 1, *p* = .018). However, none of the post hoc comparisons reached significance (at *p* < .05). Additionally, *T*‐tests revealed no differences between group 1 (short sleep duration and insomnia) and group 3 (normal/long sleep duration and insomnia); (threshold = 0.01, *p* = .917; threshold = 0.3, *p* = .591; threshold = 1, *p* = .554). We did not correct for multiple testing because none of the tests reached the 0.05 significance level.

### Correlation models

Correlations for the different groups and the total sample are displayed in Table 5. Regarding depression and insomnia, we found a slightly higher correlation for the short sleep duration group (*r* = 0.47) as compared to the other two groups (concordant normal/long sleep duration, *r* = 0.44, and discordant for sleep duration, *r* = 0.40). Model comparison showed that these three correlations could not be equated without a significant decrease in model fit (*p* = .026). Similarly, for both anxiety and anxiety sensitivity we found higher correlations with insomnia for the short sleep duration group (*r* = 0.46 and 0.42 for anxiety and anxiety sensitivity respectively) as compared to the normal/long sleep duration group (*r* = 0.38 and 0.34 for anxiety and anxiety sensitivity respectively). The discordant sleep duration group showed similar correlations (*r* = 0.38 and 0.36 for anxiety and anxiety sensitivity respectively). When these correlations were equated (among sleep duration groups for each one of the anxiety variables in turn), the models showed a significant worsening of fit. Therefore, there are statistical differences between the sleep length groups for the correlations between symptoms of insomnia and anxiety (*p* = .025) and for the correlations between symptoms of insomnia and anxiety sensitivity (*p* = .028). Finally, correlations between insomnia and educational achievement and insomnia and psychotic‐like experiences were similar across sleep length groups and not significantly different (*p* > .05). After correcting for multiple testing (0.05/5 = 0.01) none of the differences reached the significance level.

**TABLE 5:** Correlations and 95% confidence intervals between insomnia symptoms and associated variables for each sleep duration group and total sample.

	Total sample	Concordant twin pairs for short sleep duration	Concordant twin pairs for normal/long *t* sleep duration	Discordant twin pairs for sleep duration	*p* value
Anxiety symptoms	0.39 (0.37, 0.41)	0.46 (0.40, 0.51)	0.38 (0.35, 0.40)	0.38 (0.35, 0.41)	**.025**
Anxiety sensitivity symptoms	0.36 (0.34, 0.38)	0.42 (0.37, 0.48)	0.34 (0.31, 0.37)	0.36 (0.33, 0.39)	**.028**
Depression symptoms	0.43 (0.41, 0.45)	0.47 (0.42, 0.52)	0.44 (0.41, 0.46)	0.40 (0.37, 0.43)	**.026**
GCSE grades	−0.04 (−0.06, −0.01)	−0.03 (−0.11, 0.04)	−0.02 (−0.06, 0.01)	−0.06 (−0.11, −0.01)	.443
Psychotic‐like symptoms	0.26 (0.23, 0.30)	0.26 (0.16, 0.35)	0.25 (0.20, 0.29)	0.29 (0.23, 0.35)	.449

*Note*: *p* value refers to statistical differences between the three different correlations. Bold figures represent significant differences (*p* < .05). *p* value corrected for multiple testing = .01. None of the models reached the corrected significance level.

## DISCUSSION

### Summary

This study of adolescents was inspired by a theory proposed in adults (Vgontzas et al., 2013) suggesting that there may be greater genetic influence on insomnia with short sleep as compared to that with normal length/long sleep duration. We also looked at the association between these two subtypes of insomnia and a wide variety of traits which have been associated with insomnia. Overall, we provided mixed support.

First, we ran twin analyses and found no significant differences between genetic influence for insomnia with normal/long sleep duration as compared to insomnia with short sleep duration. Moreover, insomnia with short sleep duration was substantially influenced by common shared environmental factors whereas the impact of these factors was negligible for insomnia with normal/long sleep duration which is compatible with the available literature. Note that heritability is a population statistic which explains the proportion of the phenotypic variance due to genetic factors in a specific population at a specific time. Therefore, it can vary across populations (e.g., adults) and it is not informative at an individual level.

Our second set of analyses examined the theory from a molecular genetic perspective, examining PGS for insomnia symptoms and sleep duration in subjects with short and without short sleep duration. Here, we found an increase in PGS scores showing that subjects with insomnia and short sleep duration have a greater biological risk for insomnia as compared to those with normal/long sleep duration (as expected by the original theory), although these differences were not statistically significant. Of note, genetic risk can be estimated at an individual level when using PGS.

The third set of analyses examined the associations between insomnia symptoms and associated traits in those with short sleep length as compared to those without. We expected insomnia with short sleep length to have a greater association with other difficulties/poorer performance and our results supported this in part. Indeed, we found that there was a stronger association between insomnia symptoms and anxiety, and depression symptoms in those with short sleep duration as compared to those with normal/long sleep duration. These findings (although not robust once adjusting for multiple testing) support the idea that insomnia with short sleep duration could be a more severe subtype of insomnia.

### Insomnia with short sleep—A more biological origin?

In adolescents, genetic influences on insomnia with short sleep duration were lower as compared to insomnia with normal/long sleep duration. However, we note that heritability is a population statistic, meaning that different results might be obtained in different populations or in participants of different age groups. For example, a previous study found that 37% of the variance, for insomnia with short sleep duration, was explained by genetic factor (common‐shared environmental influences had a negligible impact) in a sample of adult twins (Winiger et al., 2020). We note from the literature that whereas we rarely see an influence of shared environment on insomnia symptoms or sleep length in young adults or other adults (Gregory et al., 2016; Madrid‐Valero, Rubio‐Aparicio, et al., 2020; Madrid‐Valero et al., 2021), we do find this influence in children (Fisher et al., 2012; Touchette et al., 2013). Such factors could help to explain why our results show a higher influence of environmental factors in the short sleep duration group.

Furthermore, PGS analyses are not an infallible tool and their accuracy relies on the GWAS used to calculate the PGS. Despite the fact that genetic predisposition is fixed at conception, genetic influence can change depending on factors such as age, environmental factors and previous diseases/disorders (Lewis & Vassos, 2020). Therefore, future research should aim to compare PGS in participants with and without short sleep duration in different populations (e.g., adults).

### Insomnia with short sleep—Stronger associations with other difficulties?

We tested the associations between insomnia and depression, anxiety, educational achievement and psychotic‐like experiences. Slightly higher correlations (although non‐significant differences after correction for multiple testing) were found between insomnia and depression, anxiety and anxiety sensitivity (but not educational achievement or psychotic‐like symptoms) in the short‐sleep as compared to the normal/long sleep length group. Previous publications have demonstrated that insomnia with short sleep duration has a more harmful impact on health (Anna Karin et al., 2021; Fernandez‐Mendoza et al., 2012, 2021) although for some variables this finding is inconsistent (Johann et al., 2017).

### Implications

This is the first study to test the aetiology of these different sub‐types of insomnia in adolescents. Our results showed that shared environmental factors play a major role for explaining individual differences in insomnia symptoms in those participants with short sleep duration (but not for the normal/long sleep duration group). One possibility is that the C component influencing insomnia with short sleep duration could reflect twins sharing a bedroom. This possibility needs to be tested directly in future research. We did not find a significant difference in genetic influence between the two subtypes of insomnia. Should these findings be replicated they have potential implications for clinical practice since sleep is a modifiable factor and future studies could aim to identify possible environmental risk factors to prevent the onset and/or the progression of insomnia (specially in those adolescents suffering from insomnia with short sleep duration). In this study we also used PGS which are a useful tool in predicting participants at risk of suffering insomnia. Our results show that PGS calculated using the reference GWAS for insomnia in adults (Jansen et al., 2019) are significantly associated with symptoms of insomnia in adolescence. Therefore, PGS could be a useful tool to identify individuals at risk of suffering insomnia in adolescence. However, these results must be interpreted with caution since the variance explained by PGS was limited. Furthermore, PGS did not differ significantly between the insomnia subtype groups (although results were in the expected direction). Finally, our results also showed significant associations between insomnia and anxiety/depression (with stronger associations for those with short sleep duration). This is noteworthy since treatments for insomnia also result in improvement in depressive symptoms (Ho et al., 2020) therefore, early interventions in adolescence for insomnia could also help us to prevent the onset of other disorders such as depression—and it will be informative to see whether interventions of this type are differentially effective for those with short sleep as compared to others.

### Strengths and limitations

This study has several strengths such as the theory‐driven research questions (Vgontzas et al., 2013), the use of a very large representative twin sample, and the employment of both quantitative and molecular genetic approaches. Nonetheless, results from this study must also be interpreted in light of some limitations. First, we used subjective measures to assess sleep. This is noteworthy as the original theory proposed by Vgontzas et al. (2013) clearly focuses on insomnia with *objective* short sleep duration. Unfortunately, objective measures of sleep duration were not feasible due to the resources required to assess sleep this way in the large sample needed to carry out this type of twin design. However, we acknowledge previous research highlighting discrepancies between sleep length assessed objectively and subjectively (Short et al., 2012) and note that those with insomnia are sometimes poor at assessing their sleep length (Fernandez‐Mendoza et al., 2011)—meaning that the results of this study need to be replicated using objective measures to assess sleep. Nonetheless, we consider our approach to provide useful information as significant differences have previously been found between the different types of insomnia even when sleep duration is assessed *subjectively* and this information has been flagged previously in support of the theory (Chandola et al., 2010). Furthermore, the relevance of subjective assessment is clear as insomnia is usually assessed through such measures in clinical practice.

Second, this study focused on adolescents. While this is an important age‐group given that interventions should be targeted early in life, the original theory appeared to focus primarily on adults (i.e., the studies referenced focused primarily on adults) who require a different amount of sleep to that required by adolescents. For that reason, we revised the sleep length cut‐off (to <7 h/night) given that the one suggested by the theory (<6 h/night) was not relevant to our age group. Of note the <7 h/night cut‐off that we use here has been previously used in adolescents (Fernandez‐Mendoza, Calhoun, et al., 2016). It was not possible to run sensitivity analyses using the cut‐off suggested by Vgontzas and colleagues because of the small number of participants experiencing sleep of this length (which is expected in a sample of adolescents). Future work, utilising very large samples might therefore benefit from defining short sleep length using alternative cut‐offs—and cut‐offs for sleep length subtypes need to be validated for participants of other ages in future research. Related to this point, the statistical power for some analyses (i.e., PGS) was reduced (as compared to analyses with the whole sample) due to the small number of participants in some groups (e.g., participants with insomnia and short sleep duration [*N* = 110] or participants with insomnia but with long/normal duration [*N* = 391]).

Overall, this study requires replication in samples of different ages such as adults and using different measures and cut‐offs. GWAS studies could also aim to test whether there are different genetic variants influencing insomnia with short sleep duration as compared to insomnia with normal/long sleep duration. Therefore, performing GWAS for each insomnia sub‐type could prove informative. Different covariates could also be added to future models in order to try to explain differences in terms of shared environmental influences on insomnia between sleep duration groups.

Results from this study are potentially of interest for both basic research and clinical practice. Our study highlights the major role of environmental influences in explaining individual differences for insomnia among the short sleep duration group and, consequently as a potential modifiable factor. Furthermore, this study also helps to adapt this leading theory of insomnia subtypes to an adolescent sample.

## AUTHOR CONTRIBUTIONS

Alice M. Gregory, Angelica Ronald, Melanie Schneider and Juan J. Madrid‐Valero contributed to the study conception and design. Analyses were performed by Juan J. Madrid‐Valero, Helena M. S. Zavos, Saskia Selzam and Frühling Rijsdijk. The first draft of the manuscript was written by Juan J. Madrid‐Valero under the supervision of Alice M. Gregory. All authors commented on previous versions of the manuscript and read and approved the final manuscript.

## CONFLICT OF INTEREST STATEMENT

Alice M. Gregory is an advisor for a project initially sponsored by Johnson's Baby. She is a consultant for Perrigo (2021+). She receives royalties for two books *Nodding Off* (Bloomsbury Sigma, 2018) and *The Sleepy Pebble* (Flying Eye, 2019). She has another contract with Lawrence King Publishers (publication due 2023). She was previously a CEO of *Sleep Universal LTD* (2022). She is a regular contributor to BBC Focus magazine and has contributed to other outlets (such as The Conversation, The Guardian and Balance Magazine). She occasionally receives sample products related to sleep (e.g., blue light blocking glasses) and has given a paid talk to a business (Investec). She is a specialist subject editor at JCPP (sleep) for which she receives a small honorarium. She has contributed a paid article to Neurodiem. The remaining authors have declared they have no competing or potential conflicts of interest.

## ETHICAL CONSIDERATIONS

TEDS and consent procedure were approved by the King's College London ethics committee (ref: PNM/09/10‐104).

## Supporting information

Table S1

## Data Availability

Under restrictions. Juan J. Madrid‐Valero had full access to all the data in the study and takes responsibility for the integrity of the data and the accuracy of the data analysis.
