# Supplementary material for: Sub‐types of insomnia in adolescents: Insights from a quantitative/molecular twin study
Source: JCPP Adv. 2023 May 6;3(2):e12167. doi: 10.1002/jcv2.12167 (PMC10519740; doi:10.1002/jcv2.12167)
Supplement: Supplementary file 1 — Table S1 [file JCV2-3-e12167-s001.docx]

**Supporting Information**

Table S1: Unstandardized estimates for short and normal/ long sleep duration groups.

|  | Short sleep duration | | | Normal/ long sleep duration | | |
| --- | --- | --- | --- | --- | --- | --- |
| Model | A | C | E | A | C | E |
| Full-  Heterogeneity (qualitative and quatitative differences) | 0.848 | 0.302 | 1.129 | 0.656 | 0 | 0.888 |
| **Quantitative-Heterogeneity** | **0.525** | **0.630** | **1.188** | **0.654** | **0.017** | **0.889** |
| Homogeneity | 0.810 | 0 | 0.961 | 0.810 | 0 | 0.961 |

A, additive genetic influences; C, common-shared environmental influences E, non-shared environmental influences
